# Association of immune cells with the risk of basal cell carcinoma: A bidirectional Mendelian randomized study

**DOI:** 10.1097/MD.0000000000044656

**Published:** 2025-09-26

**Authors:** Suyao Wang, Yuying Li, Shaolei Huang

**Affiliations:** a Traditional Chinese Medicine Department, People’s Hospital of Lixia District, Jinan, Shandong Province, China; b Jining Hospital of Xiyuan Hospital of CACMS, Jining, Shandong Province, China; c Acupuncture and Moxibustion Department, Affiliated Hospital of Shandong University of Traditional Chinese Medicine, Jinan, Shandong Province, China; d Shandong University of Traditional Chinese Medicine, Jinan, Shandong Province, China.

**Keywords:** basal cell carcinoma, causal relationship, GWAS, immune cells, Mendelian randomization

## Abstract

Basal cell carcinoma (BCC) is globally recognized as the most prevalent malignant skin tumor. Despite ample evidence suggesting that immune cells play a significant role in its development, the causal relationship between the 2 remains unclear. This study employed the Mendelian randomization (MR) analysis approach, utilizing publicly available genome-wide association studies data on 731 immune cell phenotypes and BCC. We primarily employ the inverse variance weighting method for our analysis, reinforced by several supplementary methodologies such as MR Egger, weighted median, simple model, and weighted model. Subsequently, we performed a comprehensive sensitivity analysis to evaluate the heterogeneity and horizontal pleiotropy effects of the findings, ensuring their robustness and reliability. According to the results of MR analysis, at a significance level of 0.01, we identified 14 immune phenotypes that were causally associated with BCC. After adjustment for multiple testing based on false discovery rate (FDR) method, 2 immune phenotypes were still significantly associated with BCC. One of them is a risk factor (*P*_FDR_ < .05, odds ratio [OR] > 1), another is a protective factor (*P*_FDR_ < .05, OR < 1). After reverse analysis, at a significance level of 0.01, we detected a causal relationship between BCC and 6 immune phenotypes. Four of them are positively correlated with the occurrence of BCC (*P* < .01, OR > 1), the other 2 are negatively correlated with the occurrence of BCC (*P* < .01, OR < 1). Nevertheless, after FDR correction, no statistically significant immune phenotypes were identified. The findings presented above suggest the lack of heterogeneity or horizontal pleiotropy (*P* > .05). In the present study, we conducted a bidirectional MR analysis to investigate the causal relationship between immune cells and BCC, unveiling the complex interaction between BCC and the immune system. In the future, the immune cells identified in this study as having a causal relationship with BCC may emerge as pivotal molecules facilitating early identification, intervention, and treatment of BCC.

## 1. Introduction

Basal cell carcinoma (BCC) is the most common malignancy among Caucasians,^[1]^ arising from the basal cell layer of the skin and accounting for about 80% of nonmelanoma skin cancers.^[2]^ BCC can cause extensive tissue destruction, leading to ulcers that extend from the epidermis to soft tissues and bone, often referred to as “invasive ulcers.”^[3]^ Chronic sun exposure is the primary risk factor, and UV-induced activation of the Hedgehog signaling pathway is considered the key pathogenic mechanism.^[4–7]^ Diagnosis is typically straightforward through skin biopsy, yet up to 10% of cases are diagnosed at an advanced stage.^[8]^ Surgery is the most common treatment, but recurrent tumors can be difficult to detect, as they may grow within scars or beneath prior surgical sites, leading to unresectable relapses. Recurrence also increases the risk of developing other skin cancers, such as squamous cell carcinoma. In rare cases, BCC can metastasize to lymph nodes or distant organs like the lungs and bones, resulting in a poor prognosis with survival ranging from 8 months to 3.6 years.^[9,10]^

Immune cells are a specialized group of cells crucial to or closely linked with immune responses, collectively known as leukocytes. The relationship between the immune system and cancer is traditionally described by the “immune editing” hypothesis,^[11]^ which includes 3 stages: *elimination*: the immune system destroys cancer cells through innate and adaptive responses; *equilibrium*: cancer cells that survive immune attack coexist with the immune system without growing; *escape*: cancer cells grow and spread unchecked due to immune system failure.^[12,13]^

Multiple studies have shown that immune cells play a bidirectional regulatory role in BCC.^[14–16]^ On the one hand, immune cells exhibit antitumor effects through immune surveillance and cytotoxic activity, promoting tumor regression.^[17,18]^ On the other hand, immune mechanisms can contribute to disease development and progression, and may even increase the risk of recurrence.^[19–21]^ Helen et al found that high levels of CD8+ T cells, interferon signaling, and IL-12/23 indicate an active antitumor immune response. In contrast, the presence of regulatory T cells, immature dendritic cells, and Th2 cytokines suggests immune suppression in BCC patients.^[22]^ Over the past few years, cancer treatments have gradually shifted from broad-acting therapies like chemotherapy and radiotherapy to more mature immunotherapies.^[23–26]^ Targeted immune checkpoint inhibitors are now capable of inducing durable antitumor responses across a range of tumor types.^[27]^ In conclusion, immune cells play a crucial role in the process of BBC. However, the existence of a causal relationship between specific subtypes of immune cells and BBC remains unknown.

Mendelian randomization (MR) is an epidemiological method for inferring causal relationships. It uses genetic variants associated with exposure factors as instrumental variables (IVs) to assess their impact on specific outcomes. By leveraging the random inheritance of genetic variants, MR avoids common limitations of observational studies, such as confounding and reverse causality, and can efficiently utilize large-scale genome-wide association study (GWAS) data. Combining MR with GWAS enables researchers to evaluate the causal link between immune cells and BCC. Although MR cannot reveal underlying molecular mechanisms (a key limitation in cancer research) it still provides valuable genetic evidence to support the development of BCC immunoprevention and therapy.

## 2. Materials and methods

### 2.1. Study design

**Figure 1.**
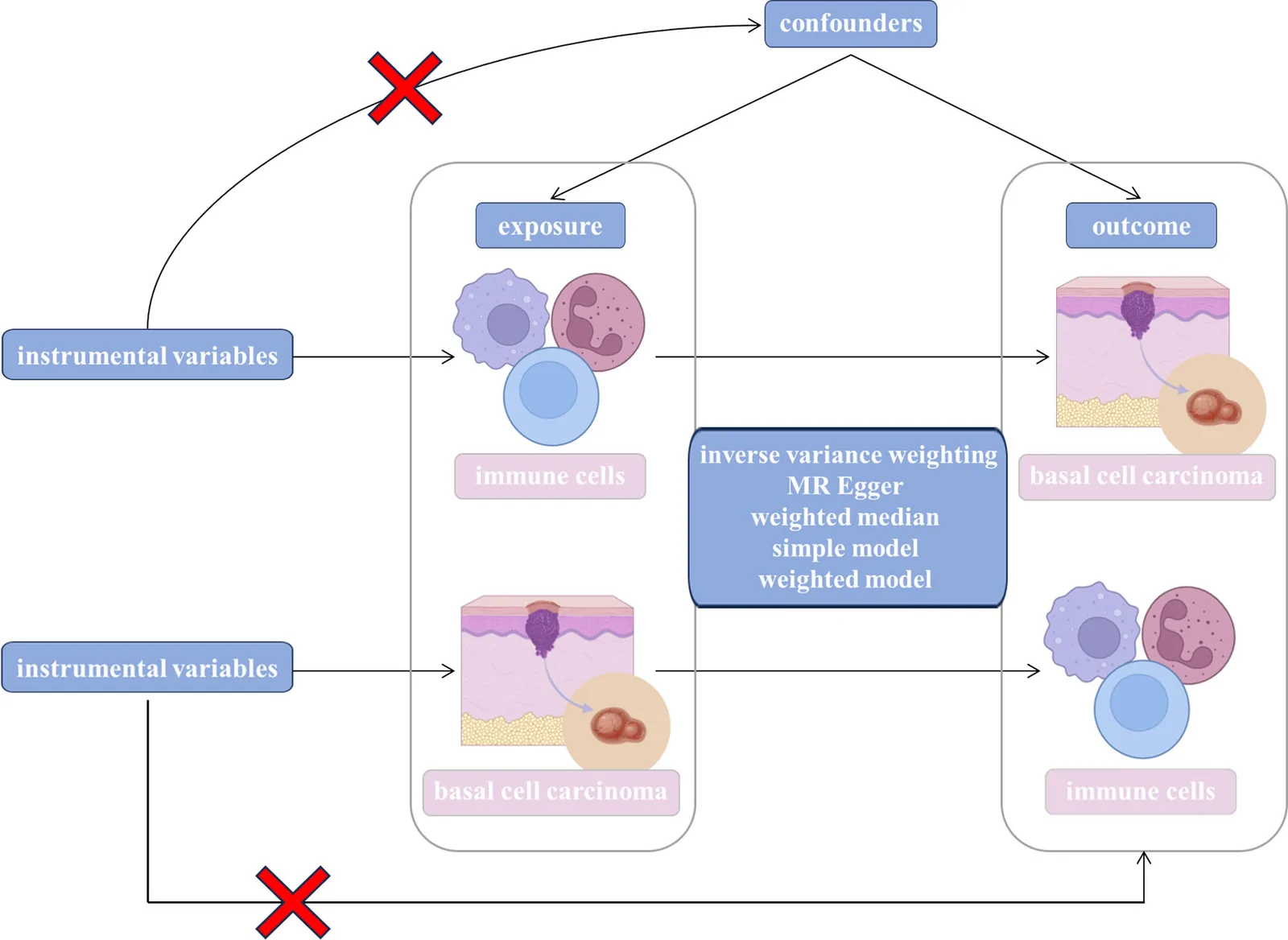
The overall design flowchart of this experiment.

In this thesis, we conducted an analysis using 2-sample MR to investigate the causal relationship between 731 immune cell phenotypes as “exposure” and BCC as “outcome.” Then, we also conducted inverse MR analysis. The IVs required for the study should satisfy the following 3 major assumptions^[28]^: exhibited a significant correlation with factors of exposure; demonstrated no association with any potential confounding variables related to both exposure and outcome; the outcome can only be influenced by exposure factors. The data utilized in this study were acquired from the publicly accessible OPEN GWAS database, which does not involve the disclosure of personal privacy or require institutional informed consent authorization. Hence, no ethical review was deemed necessary. The overall design of the experiment is shown in Figure 1.

### 2.2. Genome-wide association study data sources

The genome-wide data of BCC and immune cells were obtained from the GWAS Catalog database. The BCC data^[29]^ involved in 17,416 cases and 375,455 controls BCC patients (serial number ebi-a-GCST90013410). Immune cells data^[30]^ (serial number ebi-a-GCST0001391-ebi-a-GCST0002121) included absolute cell count (AC) (n = 118), relative cell count (n = 192), and median fluorescence intensity reflecting surface antigen levels (n = 389), morphological parameters (n = 32). These samples are from European populations and can be accessed publicly on the website (https://www.ebi.ac.uk/gwas/). Please refer to Table 1 for details.

**Table 1:** Summary of the genome-wide association studies (GWAS) included in this MR study.

Data type	Dataset	Population	Sample size	References
Basal cell carcinoma	ebi-a-GCST90013410	European	392,871	Adolphe et al^[29]^
Immune cells	ebi-a-GCST0001391-ebi-a-GCST0002121	European	3757	Orrù et al^[30]^

MR = Mendelian randomization.

### 2.3. Selection of IVs

First, based on recent studies,^[31,32]^ we incorporate instruments that satisfy the conditions with a correlation threshold of *P* < 1 × 10^−5^. Secondly, to mitigate the impact of linkage disequilibrium on the analysis outcomes, we established a stringent *r* threshold of 0.001 and considered single nucleotide polymorphism (SNP) with a minimum inter-marker distance of 10,000 kb for our investigation. The *F* statistic represents the strength of association between the SNP and the exposure, and the larger the *F* value, the higher the strength of the IV. Therefore, to avoid the potential bias arising from weak IVs, we only retained the strong IVs with an *F* statistic value ＞ 10 for further analysis.^[33]^ In MR analysis, the formula used to calculate the *F* value is: $F=\frac{R^{2}}{1-R^{2}}\times \frac{N-K-1}{K}$,^[34]^ *R*^2^ indicates the degree of IV to explain exposure: $R^{2}=2\times MAF\times \left(1-MAF\right)\times {\left(\frac{\beta }{sd}\right)}^{2}$. Where 𝑀𝐴𝐹 is the minimum allele frequency, 𝛽 is the beta from the exposure GWAS, 𝑠𝑑 is its variance (*sd* = *se* × √n), N is the total sample size of the exposure GWAS, and *K* is the number of SNPs we have screened in the exposure. Then, we further removed confounding factors related to the outcomes using the PhenoScanner V2 database.^[35]^ Next, we extracted IVs from the BCC summary statistics and then coordinated the SNPs for exposure and outcome to ensure that the effect estimate values were consistent with the same effect alleles and excluded inversion SNPs or those with incompatible alleles. Finally, the final SNPs are obtained for MR analysis.

### 2.4. Data analysis

First of all, a series of MR analyses were conducted, including MR Egger,^[36]^ weighted median,^[37]^ inverse variance weighting,^[38]^ simple model,^[39]^ and weighted model.^[40]^ One of the most effective (highest statistical power) and commonly used methods for MR studies involving multiple genetic variants is the inverse variance weighting method,^[41,42]^ which is often superior to other methods.^[43]^ After the completion of the MR analysis, further sensitivity analysis is performed on the obtained results. Cochran *Q* method is used to test heterogeneity. The intercept term of MR Egger method was assessed to determine the presence of horizontal heterogeneity, and a significant deviation from zero would indicate its existence. Furthermore, to avoid the interference of pleiotropy, which may affect multiple traits by the same genetic variation, MR PRESSO^[44]^ was used to assess the overall horizontal pleiotropy, identify and remove outliers, and finally conduct a distortion test. Based on this assessment, several SNPs exhibiting pleiotropic effects were excluded from further analysis. We employed the leave-one-out sensitivity analysis to evaluate the impact of individual SNPs on the significance of the causal effect. Additionally, taking into the issue of multiple testing, we also performed FDR correction. Based on prior research findings,^[45]^ a value of FDR <0.05 is commonly interpreted as evidence of a substantial causal association. The findings were presented using odds ratios (OR) and 95% confidence intervals. Statistical significance was determined at a threshold of *P* < .05.

All analyses in this study were performed using the “TwoSampleMR” package (version 0.5.11) within R 4.3.2 software (R Foundation for Statistical Computing, Vienna, Austria, https://www.r-project.org/).

## 3. Results

### 3.1. Causal effects of immune cells on basal cell carcinoma

**Figure 2.**
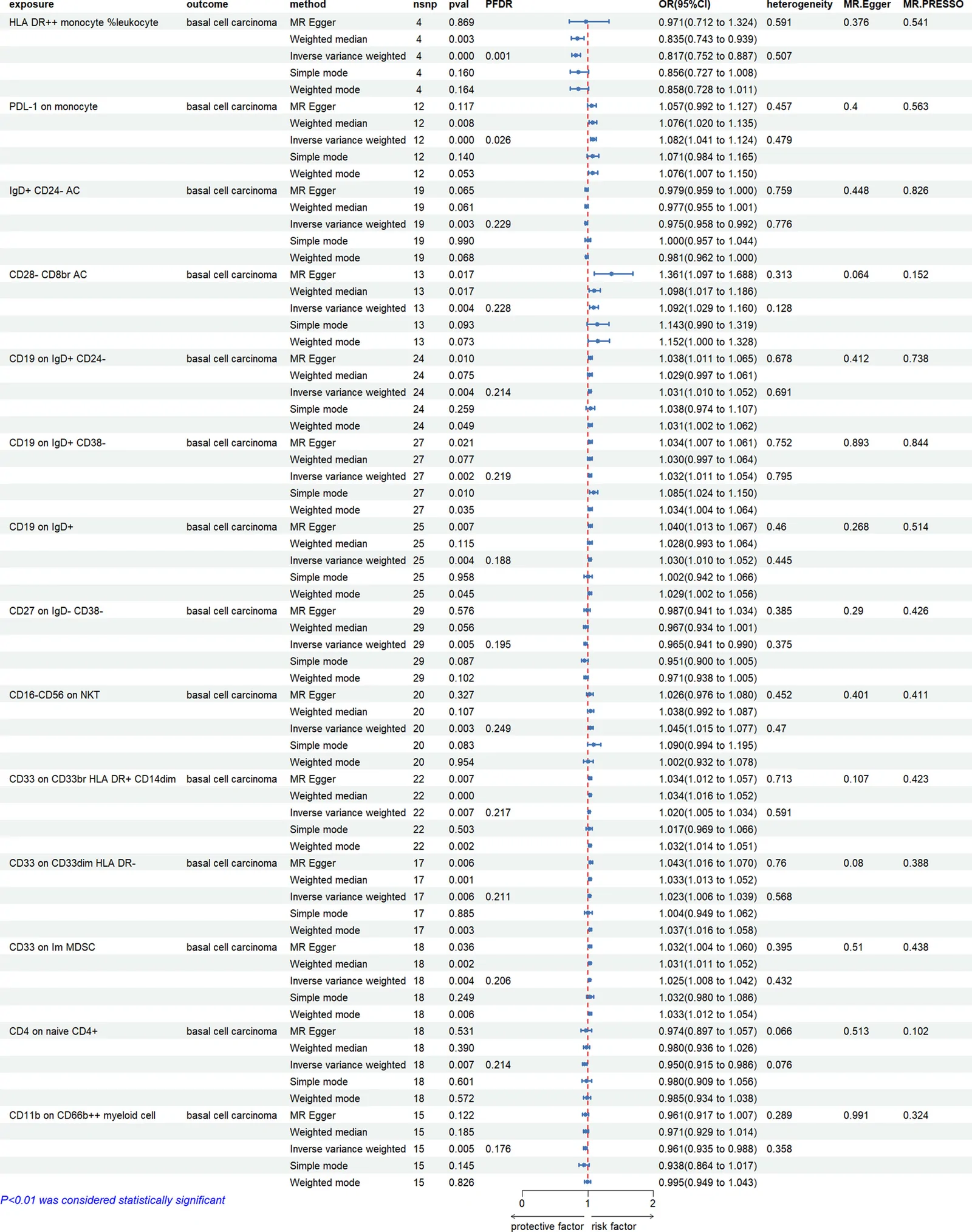
Forest plot of the causal relationship between immune cells and basal cell carcinoma.

At a significance level of 0.01, we identified 14 immune phenotypes that were causally associated with BCC. After adjustment for multiple testing based on FDR method, 2 immune phenotypes were still significantly associated with BCC (*P*_FDR_ < .05). One of them is a risk factor (OR > 1): monocyte panel: PDL-1 on monocyte (*P*_FDR_ = .026, OR = 1.082); another is a protective factor (OR < 1): T cells, B cells, natural killer cells (TBNK) panel: HLA DR++ monocyte %leukocyte (*P*_FDR_ = .001, OR = 0.817). These results indicate that even after strict FDR correction, the 2 immune phenotypes still have a relatively significant causal impact on the development of BCC. The 2 immune phenotypes respectively demonstrate their potential roles in immune suppression and antitumor surveillance. The findings from the analysis are depicted in Figure 2 and Table 2.

**Table 2:** Results of MR analysis of the causal relationship between immune cells and basal cell carcinoma.

Exposure	Outcome	Method	nsnp	pval	or	or_lci95	or_uci95
HLA DR++ monocyte %leukocyte	Basal cell carcinoma	MR Egger	4	.869	0.971	0.712	1.324
		Weighted median	4	.003	0.835	0.743	0.939
		Inverse variance weighted	4	0	0.817	0.752	0.887
		Simple mode	4	.16	0.856	0.727	1.008
		Weighted mode	4	.164	0.858	0.728	1.011
PDL-1 on monocyte	Basal cell carcinoma	MR Egger	12	.117	1.057	0.992	1.127
		Weighted median	12	.008	1.076	1.02	1.135
		Inverse variance weighted	12	0	1.082	1.041	1.124
		Simple mode	12	.14	1.071	0.984	1.165
		Weighted mode	12	.053	1.076	1.007	1.15
IgD+ CD24− AC	Basal cell carcinoma	MR Egger	19	.065	0.979	0.959	1
		Weighted median	19	.061	0.977	0.955	1.001
		Inverse variance weighted	19	.003	0.975	0.958	0.992
		Simple mode	19	.99	1	0.957	1.044
		Weighted mode	19	.068	0.981	0.962	1
CD28− CD8br AC	Basal cell carcinoma	MR Egger	13	.017	1.361	1.097	1.688
		Weighted median	13	.017	1.098	1.017	1.186
		Inverse variance weighted	13	.004	1.092	1.029	1.16
		Simple mode	13	.093	1.143	0.99	1.319
		Weighted mode	13	.073	1.152	1	1.328
CD19 on IgD+ CD24−	Basal cell carcinoma	MR Egger	24	.01	1.038	1.011	1.065
		Weighted median	24	.075	1.029	0.997	1.061
		Inverse variance weighted	24	.004	1.031	1.01	1.052
		Simple mode	24	.259	1.038	0.974	1.107
		Weighted mode	24	.049	1.031	1.002	1.062
CD19 on IgD+ CD38‐	Basal cell carcinoma	MR Egger	27	.021	1.034	1.007	1.061
		Weighted median	27	.077	1.03	0.997	1.064
		Inverse variance weighted	27	.002	1.032	1.011	1.054
		Simple mode	27	.01	1.085	1.024	1.15
		Weighted mode	27	.035	1.034	1.004	1.064
CD19 on IgD+	Basal cell carcinoma	MR Egger	25	.007	1.04	1.013	1.067
		Weighted median	25	.115	1.028	0.993	1.064
		Inverse variance weighted	25	.004	1.03	1.01	1.052
		Simple mode	25	.958	1.002	0.942	1.066
		Weighted mode	25	.045	1.029	1.002	1.056
CD27 on IgD− CD38−	Basal cell carcinoma	MR Egger	29	.576	0.987	0.941	1.034
		Weighted median	29	.056	0.967	0.934	1.001
		Inverse variance weighted	29	.005	0.965	0.941	0.99
		Simple mode	29	.087	0.951	0.9	1.005
		Weighted mode	29	.102	0.971	0.938	1.005
CD16–CD56 on NKT	Basal cell carcinoma	MR Egger	20	.327	1.026	0.976	1.08
		Weighted median	20	.107	1.038	0.992	1.087
		Inverse variance weighted	20	.003	1.045	1.015	1.077
		Simple mode	20	.083	1.09	0.994	1.195
		Weighted mode	20	.954	1.002	0.932	1.078
CD33 on CD33br HLA DR+ CD14dim	Basal cell carcinoma	MR Egger	22	.007	1.034	1.012	1.057
		Weighted median	22	0	1.034	1.016	1.052
		Inverse variance weighted	22	.007	1.02	1.005	1.034
		Simple mode	22	.503	1.017	0.969	1.066
		Weighted mode	22	.002	1.032	1.014	1.051
CD33 on CD33dim HLA DR−	Basal cell carcinoma	MR Egger	17	.006	1.043	1.016	1.07
		Weighted median	17	.001	1.033	1.013	1.052
		Inverse variance weighted	17	.006	1.023	1.006	1.039
		Simple mode	17	.885	1.004	0.949	1.062
		Weighted mode	17	.003	1.037	1.016	1.058
CD33 on Im MDSC	Basal cell carcinoma	MR Egger	18	.036	1.032	1.004	1.06
		Weighted median	18	.002	1.031	1.011	1.052
		Inverse variance weighted	18	.004	1.025	1.008	1.042
		Simple mode	18	.249	1.032	0.98	1.086
		Weighted mode	18	.006	1.033	1.012	1.054
CD4 on naive CD4+	Basal cell carcinoma	MR Egger	18	.531	0.974	0.897	1.057
		Weighted median	18	.39	0.98	0.936	1.026
		Inverse variance weighted	18	.007	0.95	0.915	0.986
		Simple mode	18	.601	0.98	0.909	1.056
		Weighted mode	18	.572	0.985	0.934	1.038
CD11b on CD66b++ myeloid cell	Basal cell carcinoma	MR Egger	15	.122	0.961	0.917	1.007
		Weighted median	15	.185	0.971	0.929	1.014
		Inverse variance weighted	15	.005	0.961	0.935	0.988
		Simple mode	15	.145	0.938	0.864	1.017
		Weighted mode	15	.826	0.995	0.949	1.043

HLA = human leukocyte antigen, MR = Mendelian randomization, OR = odds ratio.

### 3.2. Causal effects of basal cell carcinoma on immune cells

**Figure 3.**
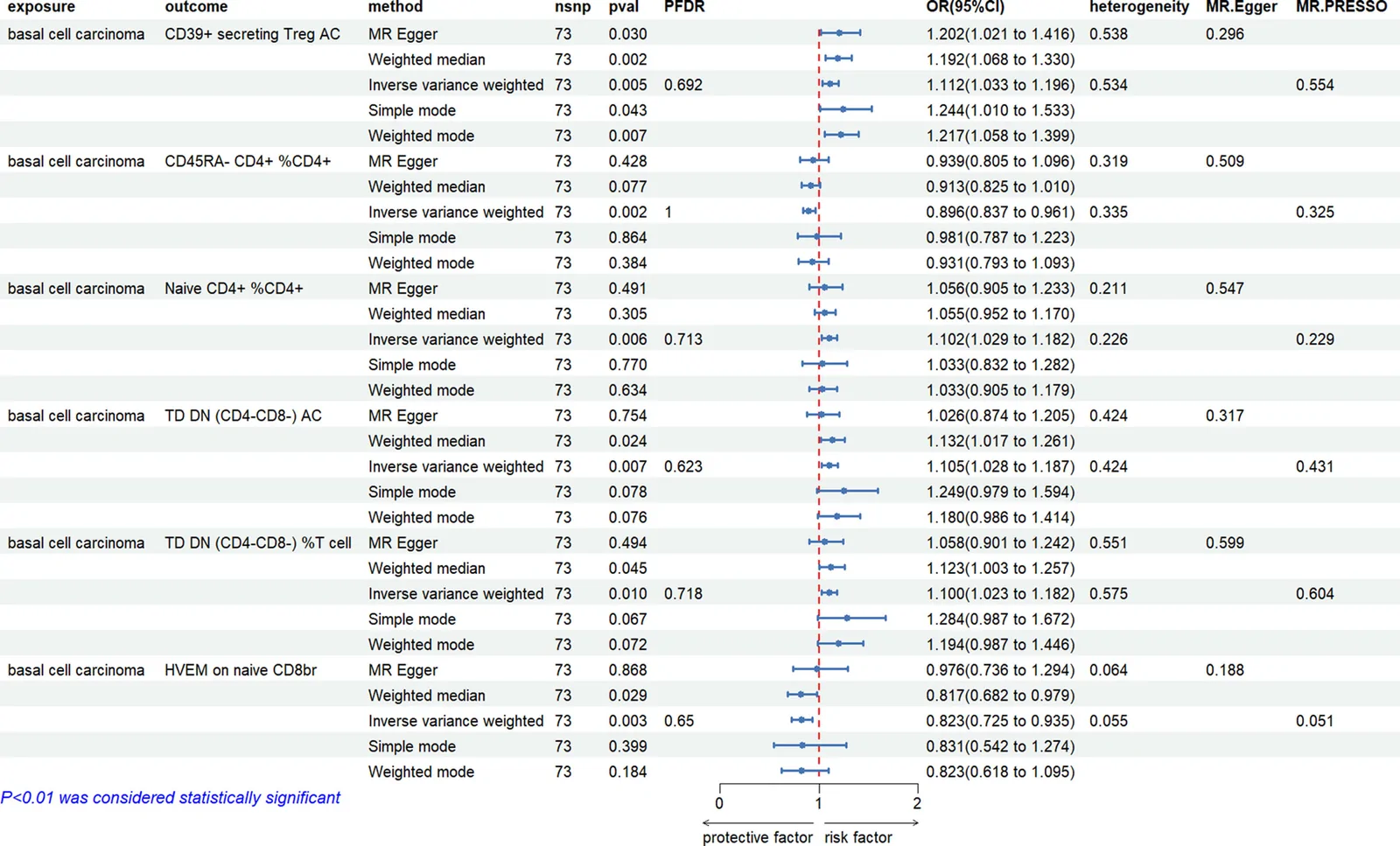
Forest plot of the causal relationship between basal cell carcinoma and immune cells.

After reverse analysis, at a significance level of 0.01, we detected a causal relationship between BCC and 6 immune phenotypes. Four of them are positively correlated with the occurrence of BCC (*P* < .01, OR > 1): Treg panel: CD39+ secreting Treg AC (*P* = .005, OR = 1.112), maturation stages of T cell panel: naive CD4+ %CD4+ (*P* = .006, OR = 1.102), TD DN (CD4−CD8−) AC (*P* = .007, OR = 1.105), TD DN (CD4−CD8−) %T cell (*P* = .010, OR = 1.100); the other 2 are negatively correlated with the occurrence of BCC (*P* < .01, OR < 1): maturation stages of T cell panel: CD45RA− CD4+ %CD4+ (*P* = .002, OR = 0.896), HVEM on naive CD8br (*P* = .003, OR = 0.823). Although the FDR-based multiple testing adjustment did not yield any statistically significant immune traits, given the exploratory nature of this study, we opted to retain the unadjusted results in order to capture as many potential positive findings as possible. Overall, these exploratory research results suggest that the progression of BCC may reshape the immune environment by regulating the quantity or function of certain T cells. Although further validation is needed, these unadjusted signals provide mechanistic hypotheses on how BCC shapes its tumor microenvironment. The findings from the analysis are depicted in Figure 3 and Table 3.

**Table 3:** Results of MR analysis of the causal relationship between basal cell carcinoma and immune cells.

Exposure	Outcome	Method	nsnp	pval	or	or_lci95	or_uci95
Basal cell carcinoma	CD39+ secreting Treg AC	MR Egger	73	.03	1.202	1.021	1.416
		Weighted median	73	.002	1.192	1.068	1.33
		Inverse variance weighted	73	.005	1.112	1.033	1.196
		Simple mode	73	.043	1.244	1.01	1.533
		Weighted mode	73	.007	1.217	1.058	1.399
Basal cell carcinoma	CD45RA− CD4+ %CD4+	MR Egger	73	.428	0.939	0.805	1.096
		Weighted median	73	.077	0.913	0.825	1.01
		Inverse variance weighted	73	.002	0.896	0.837	0.961
		Simple mode	73	.864	0.981	0.787	1.223
		Weighted mode	73	.384	0.931	0.793	1.093
Basal cell carcinoma	Naive CD4+ %CD4+	MR Egger	73	.491	1.056	0.905	1.233
		Weighted median	73	.305	1.055	0.952	1.17
		Inverse variance weighted	73	.006	1.102	1.029	1.182
		Simple mode	73	.77	1.033	0.832	1.282
		Weighted mode	73	.634	1.033	0.905	1.179
Basal cell carcinoma	TD DN (CD4−CD8−) AC	MR Egger	73	.754	1.026	0.874	1.205
		Weighted median	73	.024	1.132	1.017	1.261
		Inverse variance weighted	73	.007	1.105	1.028	1.187
		Simple mode	73	.078	1.249	0.979	1.594
		Weighted mode	73	.076	1.18	0.986	1.414
Basal cell carcinoma	TD DN (CD4−CD8−) %T cell	MR Egger	73	.494	1.058	0.901	1.242
		Weighted median	73	.045	1.123	1.003	1.257
		Inverse variance weighted	73	.01	1.1	1.023	1.182
		Simple mode	73	.067	1.284	0.987	1.672
		Weighted mode	73	.072	1.194	0.987	1.446
Basal cell carcinoma	HVEM on naive CD8br	MR Egger	73	.868	0.976	0.736	1.294
		Weighted median	73	.029	0.817	0.682	0.979
		Inverse variance weighted	73	.003	0.823	0.725	0.935
		Simple mode	73	.399	0.831	0.542	1.274
		Weighted mode	73	.184	0.823	0.618	1.095

AC = absolute cell count, MR = Mendelian randomization, OR = odds ratio.

### 3.3. Sensitivity analyses

The sensitivity analysis showed that there was no heterogeneity (Cochran *Q* test *P* > .05) or horizontal pleiotropy (MR-Egger intercept test *P* > .05) among the above immune phenotypes. The findings of MR PRESSO provide evidence against the presence of overall horizontal pleiotropy and the existence of any outliers (global test *P* > .05). Leave-one-out method and the funnel plot both indicate that the data is reliable (Figs. S1–S6, Supplemental Digital Content, https://links.lww.com/MD/Q146). Collectively, these results confirm the robustness of our causal estimates: the lack of heterogeneity shows consistent genetic effects across all instrumental variants; no pleiotropy signals (detected by MR-Egger and MR-PRESSO) support that our genetic instruments meet MR assumptions (the removal of confounding factors’ influence); sensitivity plots show no single SNP overly influences the observed associations. These multiple lines of evidence reinforce the reliability of the immune-BCC causal links.

## 4. Discussion

In this study, we employed bidirectional MR analysis for the first time to investigate the causal relationship between 731 immune phenotypes and BCC. Our study integrated large-scale GWAS datasets and systematically elucidated the genetic perspective on the role of immune cells in the pathogenesis of BCC. Our analysis provides compelling evidence that immune cells can exert an influence on the risk of BCC through a comprehensive genetic approach based on pooled GWAS data. Based on publicly available GWAS data, these 731 immune phenotypes are divided into 7 categories: B cells, myeloid cells, mature stages of T cells, TBNK, Treg, monocytes, and cDC.

In the MR analysis with immune cells as exposure and BCC as the outcome, we found that PDL-1 on monocyte in the monocyte category was a significant risk factor for the disease (*P*_FDR_ = .026), while HLA DR++ monocyte %leukocyte in the TBNK had a significant protective effect (*P*_FDR_ = .001).

PD-1 (programmed death receptor-1) is a pivotal immunosuppressive molecule, while PD-L1 functions as the primary ligand for PD-1. It exhibits widespread expression in T cells, B cells, NK cells, activated monocytes, and DC,^[46]^ thereby exerting regulatory effects on the immune function of these cell types.^[47]^ More importantly, PD-1 and PD-L1 serve as crucial immune checkpoint molecules that govern cellular apoptosis. Upon binding, they instigate the programmed death of T cells,^[48]^ impairing the cytotoxicity of immune cells against tumor cells and facilitating evasion of immune surveillance. The study conducted by Cao et al demonstrated that transgenic mice overexpressing PD-L1 in keratinocytes exhibited heightened susceptibility to developing skin tumors and an increased risk of mortality following exposure to carcinogens compared to normal mice.^[49]^ This observation may be attributed to the role of PD-L1 in keratinocytes, which aids in resolving inflammatory signaling during acute skin stress while creating conducive conditions for immune evasion and epithelial–mesenchymal transition in mutated cells. Epithelial–mesenchymal transition plays a crucial role in the biological progression of malignant tumors, enabling epithelial-derived cells to acquire enhanced migratory and invasive abilities. Hence, it can be inferred that PD-L1 acts as a contributing factor to an elevated susceptibility to skin tumors. Lipson et al demonstrated the significant expression of the above 2 molecules in BCC,^[50]^ and Malinga et al also found that the concentration of soluble PD-1 and PD-L1 in the plasma of BCC patients was statistically significantly increased,^[51]^ indicating that the body has widespread immune suppression,^[52]^ which may allow cancer cells to evade immune surveillance^[53]^ and results in the persistence and progression of tumors. Additionally, during the formation of BCC, malignant tumor tissue rapidly proliferates, leading to a highly swollen volume and an incomplete vascular system within the tumor tissue, which frequently falls short in fulfilling the rising need for oxygen in swiftly developing tumors, leading to the creation of a hypoxic setting within the tumor.^[54]^ PD-L1, as an important medium expressed in hypoxic tumor cells, has an important effect on their growth.^[46]^ This is consistent with our MR study that the risk of developing the disease increases with the proportion of PDL-1 on monocyte cells.

Instead, our study found that the risk of BCC decreased as the percentage of HLA DR++ monocyte %leukocyte cells in the TBNK increased. Human leukocyte antigen (HLA) is a product of the major histocompatibility complex in humans, and the HLA gene exhibits a complex polygenic structure with “hyperpolymorphism.”^[55]^ HLA-DR, the most classic molecule among HLA class II molecules, serves as a reliable indicator of monocyte activation status in acute pathological conditions.^[56]^ Kohchiyama et al conducted an immunohistochemical analysis on 8 BCC patients using a series of monoclonal antibodies. Their findings suggest that the expression of HLA-DR antigen on tumor cells may play a role in initiating or stimulating a cellular immune response against tumor cells through the induction of an autoimmune response, thereby representing one of the defense mechanisms employed by BCC to counteract tumor cell proliferation.^[57]^ Furthermore, McCaw et al discovered that the presence of HLA II class molecules can enhance the local activation of CD4 T cells.^[58]^ This indirect mechanism aids in promoting the activation and proliferation of CD8 T cells, thereby delaying T cell exhaustion and restraining tumor cell growth.^[59,60]^ In addition, several studies have shown that HLA class II antigens are highly expressed in tumor cells as favorable prognostic markers.^[61–63]^ In summary, the uniqueness of this immunophenotype is reflected in its dual role in BCC: regulating the tumor microenvironment to induce autoimmunity and serving as a circulating biomarker indicating impaired immune surveillance. This is consistent with the protective effect of HLA DR++ monocyte %leukocyte that we found in the MR study.

In the reverse analysis with BCC as the exposure and immune cells as the outcome, we found that the affected immune cells were mainly concentrated in T cells. According to the MR results, as the disease progresses, the CD39+ secreting Treg AC in Treg and Naive CD4+ %CD4+, TD DN (CD4−CD8−) AC, TD DN (CD4−CD8−) %T cell in mature stages of T cells showed a positive causal relationship with disease. Meanwhile, CD45RA− CD4+ %CD4+, HVEM on naive CD8br in mature stages of T cells exhibits a causal relationship with the disease that is negative.

In the tumor microenvironment of BCC lesions, prominent cellular constituents encompass cancer-associated fibroblasts, tumor-associated macrophages, and a plethora of tumor-infiltrating lymphocytes comprising CD4+ T cells, CD8+ T cells, and Tregs. According to the distinct surface-expressed differentiation antigens of cells, T cells can be classified into 2 major subpopulations: CD4+ and CD8+. Activated CD4+ T cells predominantly undergo differentiation into helper T cells, while CD8+ T cells primarily differentiate into cytotoxic T lymphocytes. A large number of preclinical and clinical studies have established that CD4+ T cells within tumors have cytotoxic programs and can directly kill cancer cells,^[64]^ while cytotoxic T lymphocytes, which are derived from CD8+ T cells, have been extensively validated for their pivotal role in antitumor immunity.^[65]^ Therefore, during the early stage of BCC, which is the elimination phase of immune editing,^[11]^ the body recognizes abnormal tumor cells, and T cells immediately initiate an immune response, leading to high expression of Naive CD4+ %CD4+,TD DN (CD4−CD8−) AC,TD DN (CD4−CD8−) %T cell to exert antitumor effects. During the subsequent equilibrium and escape stages,^[11]^ Treg, as a type of cell used to control autoimmune reactions within the body, exert highly immunosuppressive functions, thereby creating a body environment that promotes tumor progression.^[22]^ Omland et al confirmed that although Tregs are involved in cancer progression, CD39+ secreted by Tregs is specifically overexpressed in peritumoral skin compared to normal skin.^[20,66]^ This suggests that CD39+ may regulate the tumor immune response. Reverse MR analysis further supports that BCC can increase the abundance of CD39+ secreting Treg AC.

Previously, similar studies have investigated the causal associations between immune cells and skin cancer. Yin et al assessed the causal relationships between immune cells and 4 distinct types of skin cancer.^[67]^ While their study encompassed a broader range of outcomes compared to ours, our analysis incorporated a reverse MR approach, and the application of a bidirectional MR framework strengthened the reliability of causal inference. Fu et al conducted a causal relationship analysis of circulating inflammatory proteins and BCC.^[68]^ Unlike our study, their analysis was based on the levels of inflammatory proteins in plasma, while our study was based on the genetic level of immune phenotypes. Our research has the following several advantages: firstly, in comparison to randomized controlled trials, MR studies offer advantages in terms of speed and cost-effectiveness due to their utilization of existing large-scale GWAS data. This enables the exploration of potential causal relationships between modifiable risk factors and rare diseases that often necessitate extensive sample sizes and prolonged follow-up periods for adequate outcomes in randomized controlled trials.^[41]^ Secondly, since the sample size of the dataset we have chosen is large enough, it is statistically significant. Finally, we employed a wide variety of MR analysis techniques to mitigate potential confounding factors and performed sensitivity analyses using multiple approaches, thereby ensuring the strength and dependability of our conclusions.

Naturally, it is crucial to recognize the constraints of this research. Firstly, our findings are based on a European population database, thus we cannot guarantee that our findings are applicable to other ethnic groups, particularly considering the higher incidence of the disease among white individuals. Secondly, FDR correction in reverse MR analysis reduces statistical power and may mask true causal relationships. However, the unadjusted results (*P* < .01) are exploratory, and we hope to discover as many potential positives as possible for validation in larger cohorts in the future. Thirdly, in statistics, it is generally considered that |log (OR)| < 0.1 (i.e., OR between 0.91 and 1.10) represents a small effect, and an OR approximately equal to 1 indicates a weak independent effect of the exposure factor, thereby limiting clinical relevance. The reasons for this might be due to the influence of confounding factors, measurement errors, or indirect effects of exposure on the disease. Finally, although MR analysis establishes causal relationships between variables, statistical significance (a significant *P* value) does not equate to clinical significance. Our findings are entirely based on genetic inferences. Therefore, we not only need to focus on expanding the sample size to verify these findings but also conduct clinical or biological validation to further explore whether these immune cells affect the disease through their interactions within the microenvironment of BCC.

## 5. Conclusion

This study employed bidirectional MR analysis to systematically explore the causal relationship between immune phenotypes and BCC at the genetic level. The research found that PDL-1 on monocyte may act as a novel risk factor, mediating the immune escape process; while HLA DR++ monocyte %leukocyte may exert a protective effect by enhancing immune surveillance. Reverse analysis further revealed that the BCC microenvironment may drive T cell functional imbalance. We hope these findings can provide new ideas for the precise prevention and control of BCC: for instance, in early screening, specific immune cell levels can be used as noninvasive biomarkers to assist in identifying high-risk individuals; or targeted intervention measures such as selecting targeted immunotherapy drugs can be adopted to delay or even prevent the progression of cancer. Future research still requires more experimental studies to verify the mechanism and clinical application potential of specific immune phenotypes as therapeutic targets, thereby expanding the broad prospects of BCC immunotherapy.

## Acknowledgments

The authors would like to acknowledge the GWAS catalog (https://www.ebi.ac.uk/gwas/) for providing invaluable site summary data, which played a pivotal role in the inception of this study.

## Author contributions

**Conceptualization:** Suyao Wang.

**Formal analysis:** Suyao Wang.

**Funding acquisition:** Shaolei Huang.

**Methodology:** Suyao Wang.

**Software:** Yuying Li.

**Supervision:** Shaolei Huang.

**Validation:** Yuying Li.

**Writing – original draft:** Suyao Wang, Yuying Li.

**Writing – review & editing:** Shaolei Huang.

## Supplementary Material
