# Supplementary material for: Association of immune cells with the risk of basal cell carcinoma: A bidirectional Mendelian randomized study
Source: Medicine (Baltimore). 2025 Sep 26;104(39):e44656. doi: 10.1097/MD.0000000000044656 (PMC13593114; doi:10.1097/MD.0000000000044656)
Supplement: Supplementary file 1 [file medi-104-e44656-s001.pdf]

Association of immune cells with the risk of basal cell carcinoma: a bidirectional Mendelian randomized study

Supplementary Material 1

Scatter plots between immune cells and basal cell carcinoma.

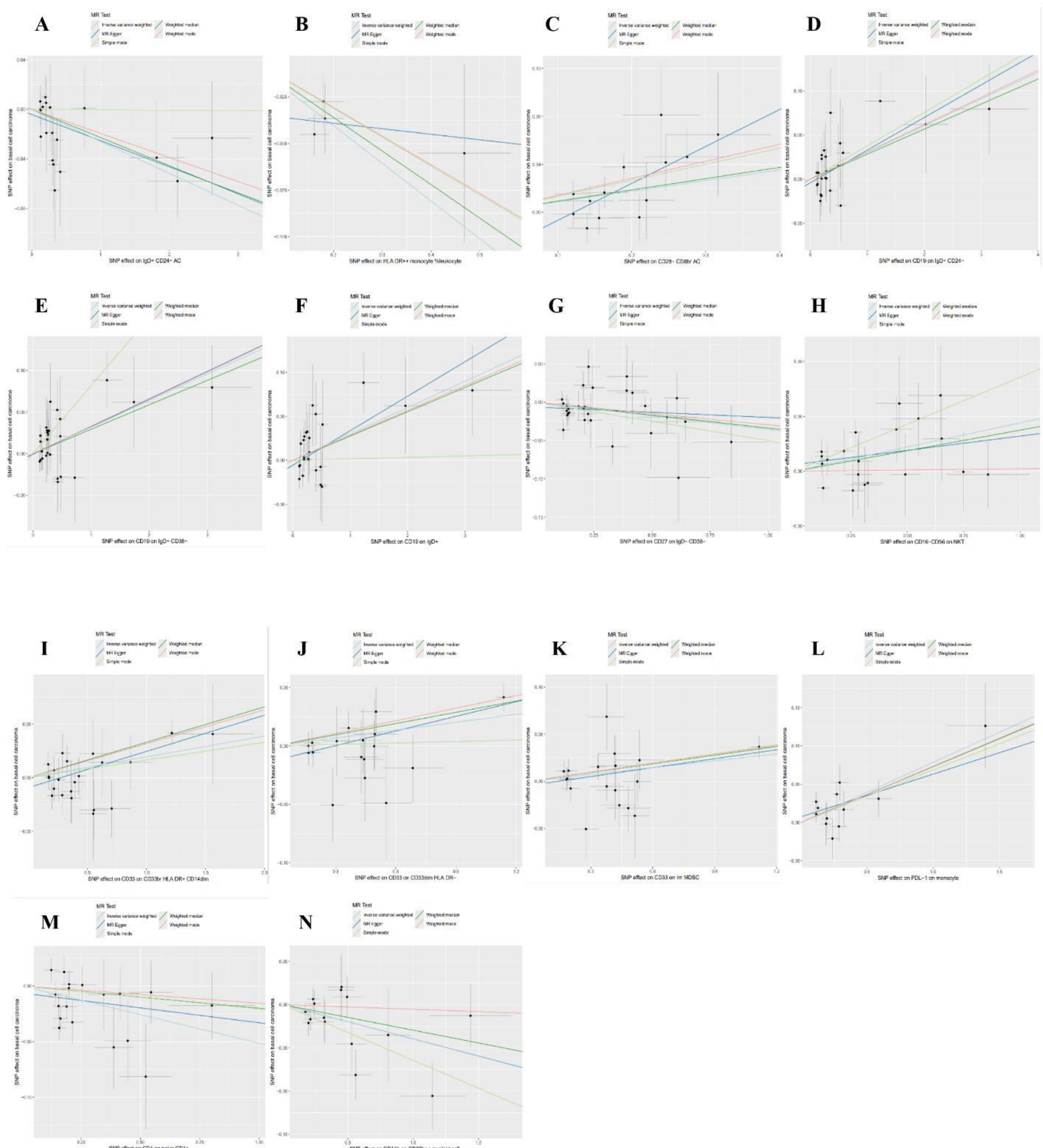

(A. IgD+ CD24- AC; B. HLA DR++ monocyte %leukocyte; C. CD28- CD8br AC; D. CD19 on IgD+ CD24-; E. CD19 on IgD+ CD38-; F. CD19 on IgD+; G. CD27 on IgD- CD38-; H. CD16-CD56 on NKT; I. CD33 on CD33br HLA DR+ CD14dim; J. CD33 on CD33dim

HLA DR-; K. CD33 on Im MDSC; L. PDL-1 on monocyte; M. CD4 on naive CD4+; N. CD11b on CD66b++ myeloid cell)

Association of immune cells with the risk of basal cell carcinoma: a bidirectional Mendelian randomized study

Supplementary Material 2

Leave-one-out method plots between immune cells and basal cell carcinoma.

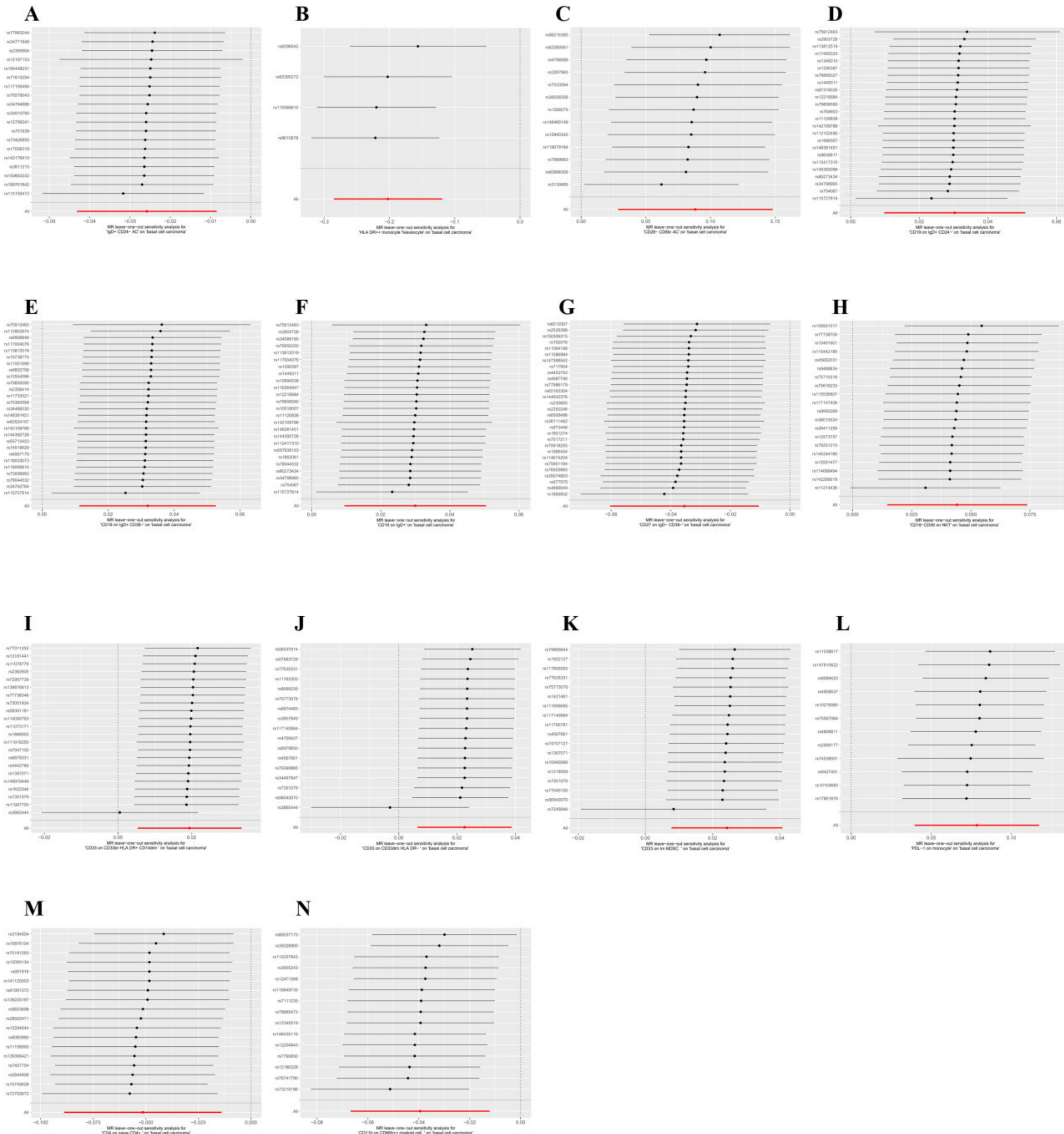

(A. IgD+ CD24- AC; B. HLA DR++ monocyte %leukocyte; C. CD28- CD8br AC; D. CD19 on IgD+ CD24-; E. CD19 on IgD+ CD38-; F. CD19 on IgD+; G. CD27 on IgD- CD38-; H. CD16-CD56 on NKT; I. CD33 on CD33br HLA DR+ CD14dim; J. CD33 on CD33dim HLA DR-; K. CD33 on Im MDSC; L. PDL-1 on monocyte; M. CD4 on naive CD4+; N. CD11b on CD66b++ myeloid cell)

Association of immune cells with the risk of basal cell carcinoma: a bidirectional Mendelian randomized study

Supplementary Material 3

Funnel plots between immune cells and basal cell carcinoma.

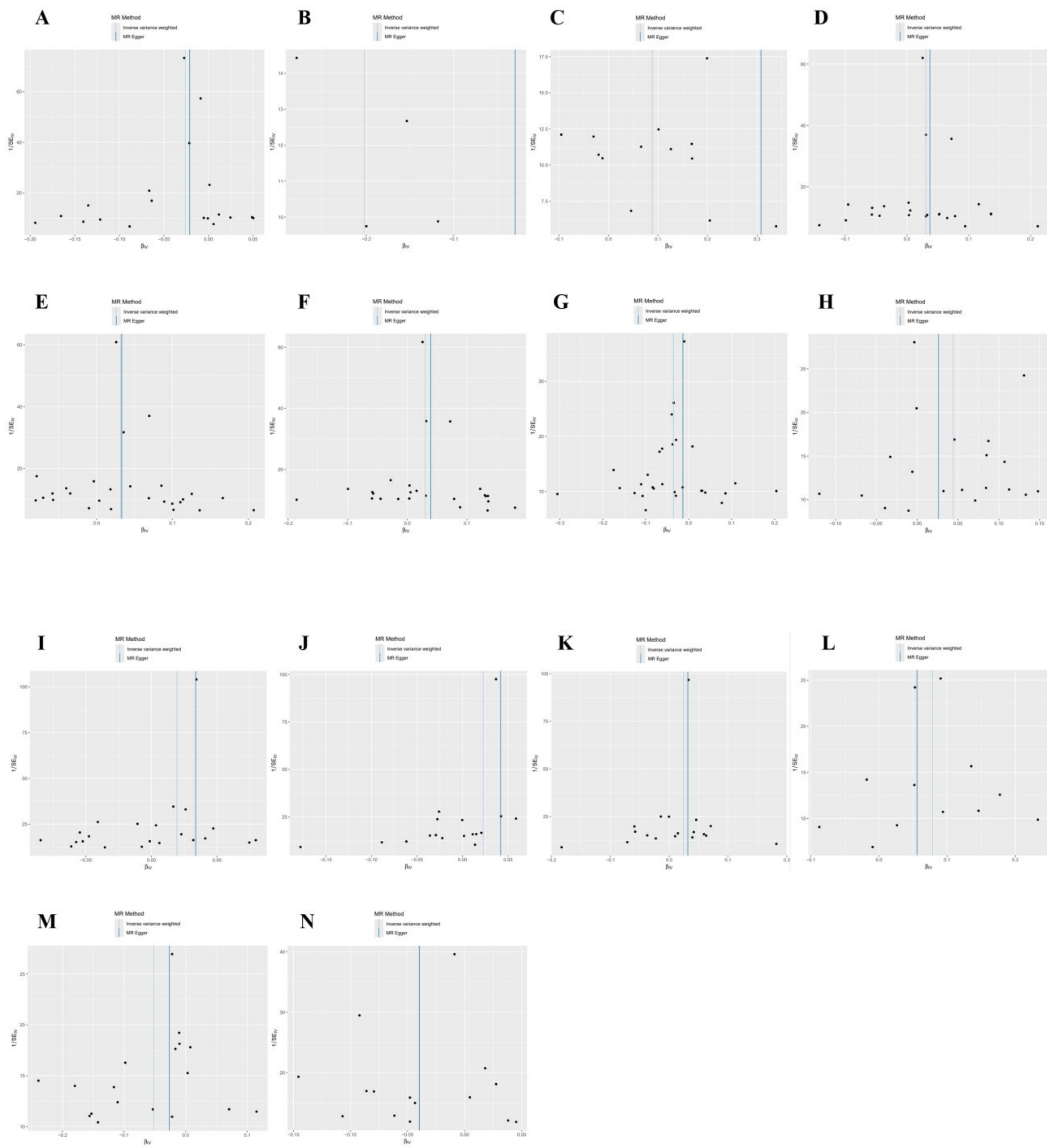

(A. IgD+ CD24- AC; B. HLA DR++ monocyte %leukocyte; C. CD28- CD8br AC; D. CD19 on IgD+ CD24-; E. CD19 on IgD+ CD38-; F. CD19 on IgD+; G. CD27 on IgD- CD38-; H. CD16-CD56 on NKT; I. CD33 on CD33br HLA DR+ CD14dim; J. CD33 on CD33dim

HLA DR-; K. CD33 on Im MDSC; L. PDL-1 on monocyte; M. CD4 on naive CD4+; N. CD11b on CD66b++ myeloid cell)

Association of immune cells with the risk of basal cell carcinoma: a bidirectional Mendelian randomized study

Supplementary Material 4
Scatter plots between basal cell carcinoma and immune cells.

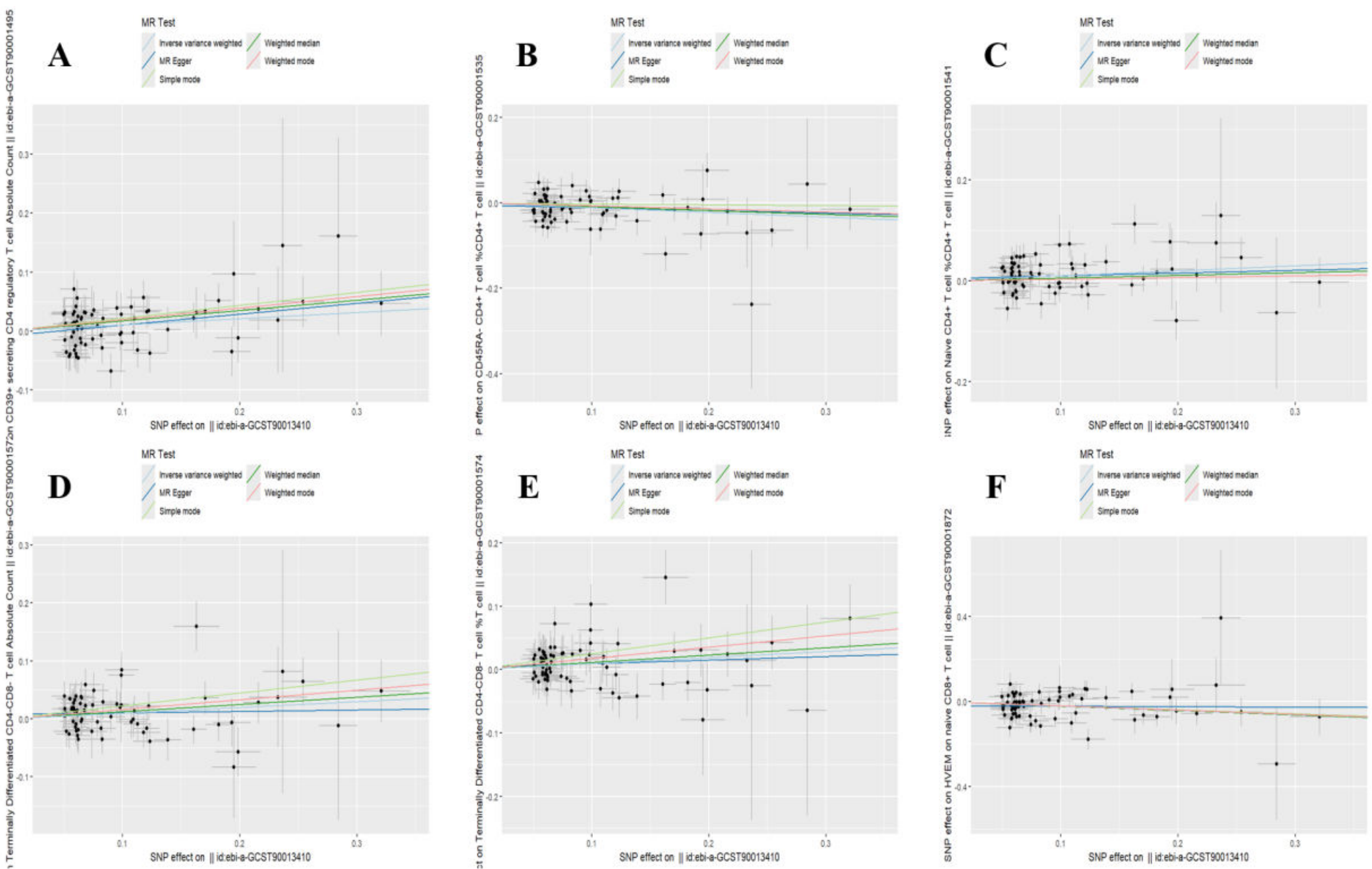

(A. CD39+ secreting Treg AC; B. CD45RA- CD4+ %CD4+; C. Naive CD4+ %CD4+; D. TD DN (CD4-CD8-) AC; E. TD DN (CD4-CD8-) %T cell; F. HVEM on naive CD8br)

Association of immune cells with the risk of basal cell carcinoma: a bidirectional Mendelian randomized study

Supplementary Material 5

Leave-one-out method plots between basal cell carcinoma and immune cells.

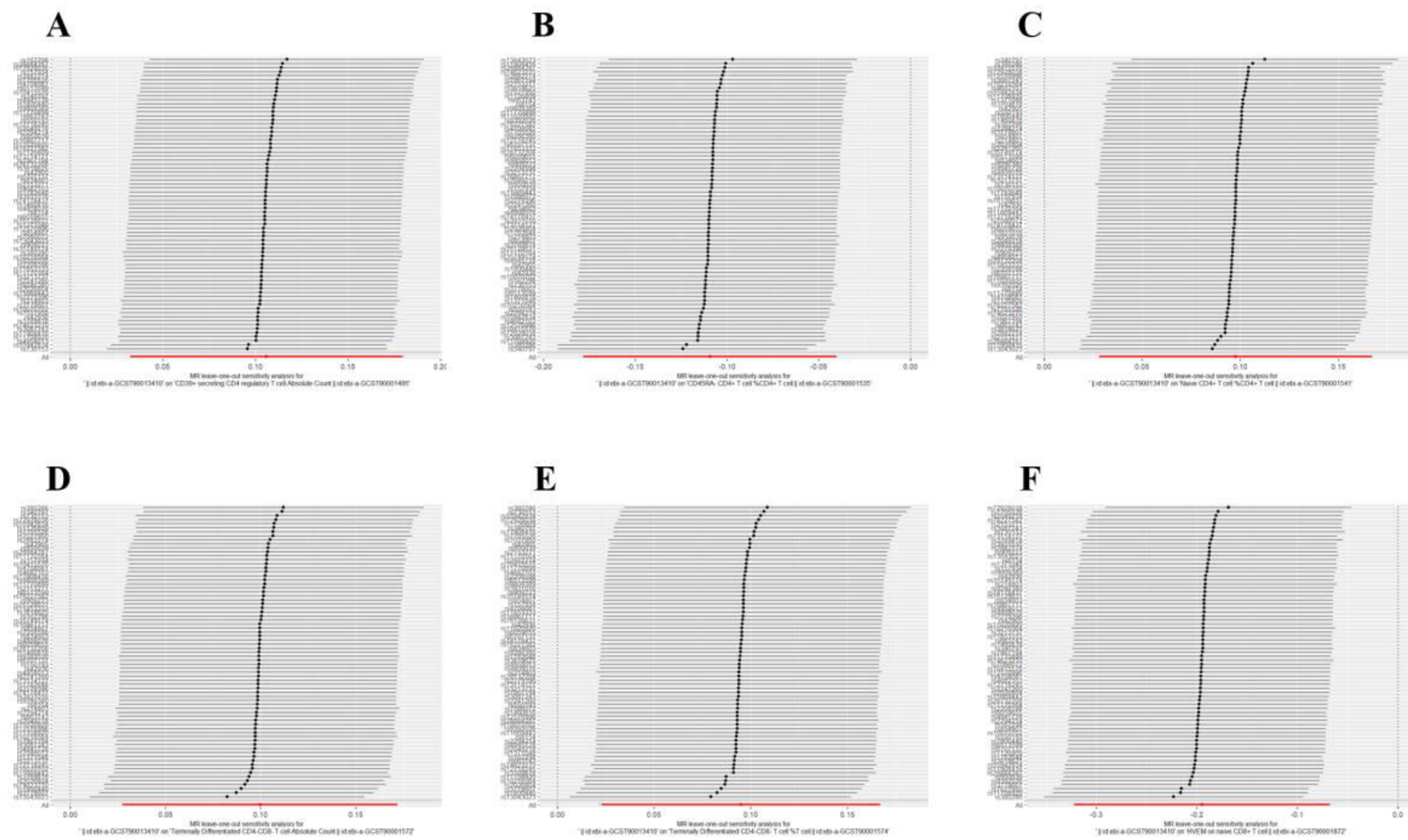

(A. CD39+ secreting Treg AC; B. CD45RA- CD4+ %CD4+; C. Naive CD4+ %CD4+; D. TD DN (CD4-CD8-) AC; E. TD DN (CD4-CD8-) %T cell; F. HVEM on naive CD8br)

Association of immune cells with the risk of basal cell carcinoma: a bidirectional Mendelian randomized study

Supplementary Material 6

Funnel plots between basal cell carcinoma and immune cells.

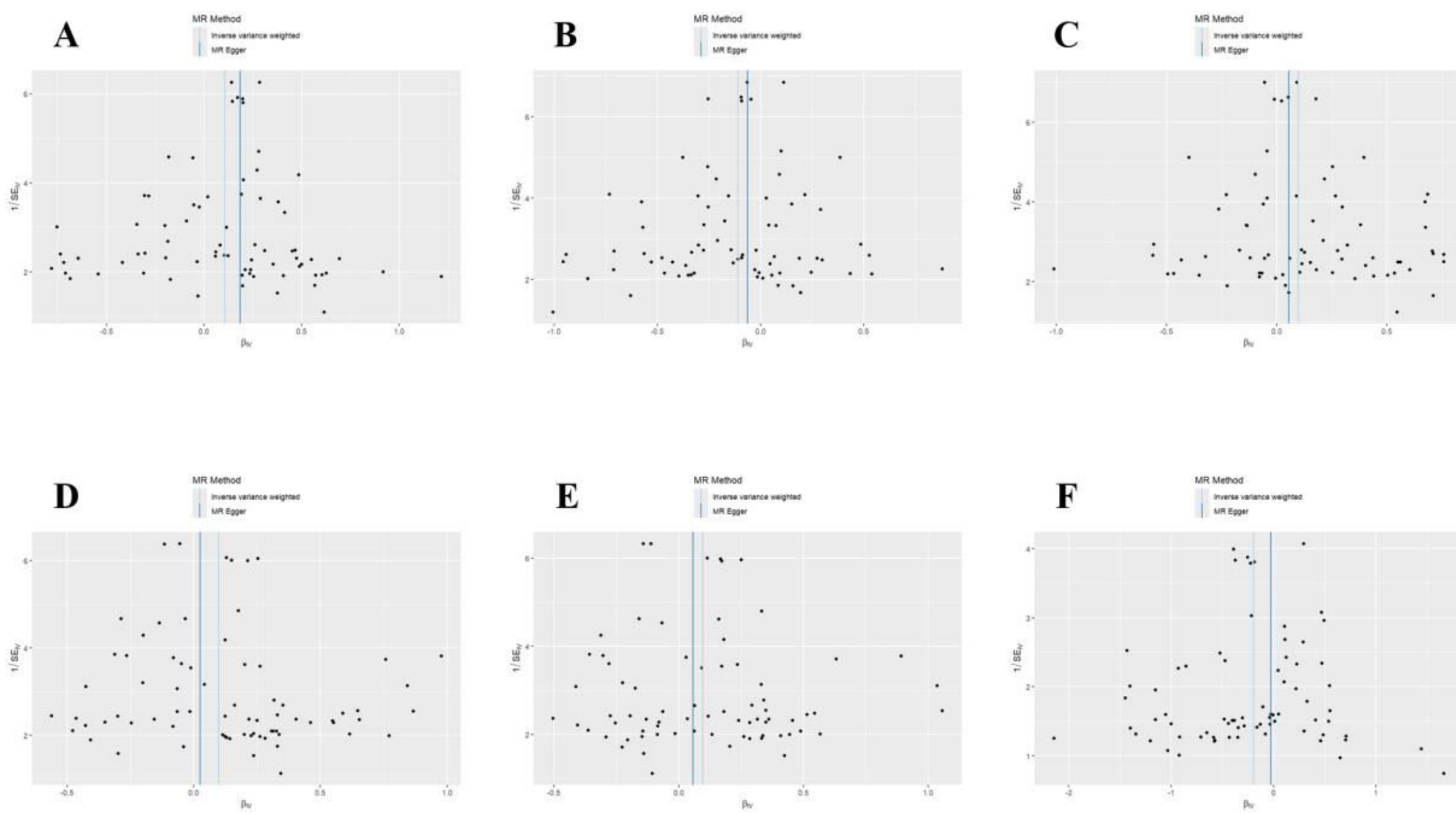

(A. CD39+ secreting Treg AC; B. CD45RA- CD4+ %CD4+; C. Naive CD4+ %CD4+; D. TD DN (CD4-CD8-) AC; E. TD DN (CD4-CD8-) %T cell; F. HVEM on naive CD8br)
